# Case of Abdominal Dropsy, in Which an Attempt Was Made to Cure the Disease by Exciting Peritoneal Inflammation

**Published:** 1826-04

**Authors:** H. R. Oswald


**Art. II.**—*Case of Abdominal Dropsy, in which an Attempt was made to cure the Disease by exciting Peritoneal Inflammation.* By H. R. Oswald, Esq.

In the month of November, 1822, Betty Craine, æt. twenty-four, a middle-sized woman, of dark leaden complexion, of Celtic parents, and phlegmatic temperament, applied to me on account of an enormous abdominal dropsy, which measured nearly six feet in circumference.

The abdomen felt exceedingly hard and tense, but was not tender. She could hardly walk across her cabin, from dyspnœa and debility, and the weight and tension of the tumor, which caused her to bend the body much forward, leaning her hands on her knees. The emaciation was very considerable; the appetite good; thirst considerable; tongue clean; pulse 120, and small; skin dry, harsh, and rough; bowels habitually costive; urine scanty.

This affection commenced about twelve months ago, after an obstruction of the catamenia for nearly a year, arising, as was supposed, from exposure to cold. The swelling was preceded by lancinating pains in the abdominal and lower part of the thoracic cavities, but which, after a few months, ceased entirely; and the disease had, in a chronic manner, gradually arrived at its present oppressive form.

Paracentesis abdominis being urgently indicated, after the exhibition of two doses of aperient medicine, it was performed on the 25th of November, 1823, in the usual manner, between the margin of the os ilium and the umbilicus, in the presence of Mr. Holford Watts and Mr. William Oswald, then my pupils, and *thirty-five quarts and a half* of clear limpid serum evacuated. We abstracted this large quantity of water gradually, by frequently stopping the discharge, and exhibiting a little spirits and water, keeping up an attention to pressure on the abdomen as it flowed. The patient bore the operation well. After the removal of the fluid, the trunk of the body presented a singular appearance. The loose integuments of the abdomen were so extensive, as to be capable of being laid to one side nearly across the lumbar region, and were thickened into a horny consistence at those points on which the patient had been in the habit of resting the tumor on any support before her; whilst the cartilages of the ribs remained projecting, according to the form and position she had assumed by becoming accommodated to the great size of the abdominal tumor.—An opiate was administered at bed-time.

November 27th.—Complained of slight pain in the head, together with tenderness in the abdomen and lumbar region. Pulse quick and small, with considerable heat of surface. Venesection was performed, and a purgative administered, with decided relief to these symptoms; and in a few days she began the use of a weak mercurial camphorated liniment, rubbed into the abdomen every night and morning, with the view of altering the action of the torpid vessels of the peritoneum. A course of diuretic and turpentine medicines, given three times a-day, with purgatives occasionally, was at the same time prescribed. As much attention to proper regimen and nursing was also recommended, as the poor circumstances in which she was placed would permit.

Though this plan was closely followed up, with various modifications, and so as to affect the mouth a little, the abdominal tumor began again to increase rapidly, and continued to do so, notwithstanding the employment of a variety of remedies usual in such cases, but which it appears to me unnecessary to detail. Care was taken that the treatment should not reduce the system; and, although it had no effect in checking the re-accumulation of the dropsy, yet the general appearance and health of the patient were manifestly improved by it. The fluid increased so rapidly, that it became necessary to repeat the operation in February 1823, little more than ten weeks after the first tapping; and twenty quarts of a dense and albuminous fluid were evacuated, very different from the limpid serous produce formerly obtained. On cooling, this fluid deposited a heavy sediment, but of which I did not examine the chemical formation. Was this change from a limpid serum to a dense albuminous fluid in so short a period, the consequence of the action of the mercury? It certainly happens in ill-conditioned ulcers and membranes, that a change from an ichorous to a healthy discharge is occasioned by the action of this mineral on the system. Or was this difference in the fluids owing to their being the contents of two different hydatids, or cysts, enlarged rapidly?

After this second operation, repeated venesection, sudorifics, and the exhibition of the compound powder of jalap, as an aperient diuretic, were practised, in conjunction with a course of steel, and occasional blistering to the affected region. These remedies all agreed pretty well with the patient; but the operation became again necessary in April, when nineteen or twenty quarts of a still more viscid fluid were discharged.

But the patient's health was now very considerably improved; for, within two days after the tapping, she was enabled to walk home (three miles and a half) without difficulty.

Excepting the purgatives, all the other remedies were now discontinued, and I put the patient upon a course of diluted arsenical frictions into the abdomen, with the view of bracing and changing the action of the system of diseased vessels.

I did not see my patient again, however, till July 24th, 1823, eight months after the first and nearly three after the third operation, when she again presented herself, labouring under a return of the disease. Her general health was very much improved, and she had gained so much flesh and muscular vigour, that, had it not been for the large size of the tumor, and the dyspnœa occasioned by it, she said she would have felt quite well. Her appetite was good; but she complained of being unable to satisfy it, on account of the great oppression of respiration occasioned by taking even a small quantity of food. Tapping was immediately performed, and twenty-two quarts of a colourless albuminous fluid discharged. The integuments and parietes of the belly now presented little induration or thickening, but were, on the contrary, flaccid and soft.

On this occasion, I reasoned in the following manner. In consequence of the failure of all the remedies tried in the cure of this case, and under the conviction that the internal surface of the abdominal sac, instead of a serous, had passed into a membrane secreting a very different fluid, in this manner rendering the disease in some degree a local one, am I warranted in attempting to produce a change in the vessels of this secreting surface, by means of a practice suggested some years previously by a case, which I shall here concisely relate?

In October, 1817, I was called to see a Mr. W—, a prisoner for debt in Castle Rushen, who laboured under dropsy. He was between fifty and sixty years of age, one who is generally described as a broken-down constitution, and a free liver, who had undergone a great deal of fatigue and various hardships in his time; and even his countenance presented a bloated and sallow aspect. He was unable to lie down, from orthopnea. General anasarca had supervened to ascites, so that, from the toe to the axilla and epigastrium, the integuments pitted deeply on pressure; and, in the legs and scrotum, the swelling was so great as to render the skin tense and shining. The abdomen was as tense as a drum, but by no means protuberant in proportion. His spirits were good; and, though his appetite admitted of little nourishment, he had supported himself, he said, by a plentiful allowance of liquor, which he could not do without, and which he could not be prevailed on to give up. Under these circumstances, tapping appeared to me to afford the best chance of relief; and it was accordingly performed, by puncturing midway between the ilium and umbilicus. The quantity of serum discharged did not amount to two gallons. The operation relieved the tension of the abdomen, of course, but the general anasarca remained in *statu quo.* As I resided ten miles distant from this patient, I left him to the care of Mr. Jones, resident practitioner of Castletown, and did not see him again for nearly a week; when I found that the wound made in the abdominal parietes had not only not closed, but looked angry and callous, and discharged water in large quantities. When I cut through the œdematous integuments, they discharged more water than blood, which caused me to suspect the puncture would be slow in healing; a circumstance not to be wondered at, considering the man's mode of living. From the continual oozing of water from it, the belly became more and more flaccid, and the general dropsy had undergone a corresponding improvement; from which circumstances, and the deep appearance of the wound, there was much reason to conclude the peritoneum remained divided; but the patient complained only of slight pain or tenderness on pressure. However, suspecting that this accident might prove the cause of serious inflammation of the peritoneum, I refrained from probing it, and decided upon letting the case take its course. I therefore confined the practice to saline diuretics and purgatives, and prescribed venesection if the pain in the abdomen on pressure should increase. He drank at this time a pint of gin daily, and denied the possibility of living without it; and, under the circumstances, I did not consider it prudent to contend with him about it. I know this concession will expose me to the censure of some, but the result proved that it was not a fatal error, however much opposed to the best-established theories and opinions. The wound continued open for upwards of a month, and closed gradually. There was no return of the ascites. The anasarca so far abated, that it was reduced to his feet and ankles; and in the autumn of 1818, the year following, I met my patient in the streets of Douglas, a tall, broken-down looking man, but as well, if not better, than he had been for some years previous to his attack of dropsy. He soon after left this island, since which I have lost sight of him.

Independent of the relief to the system arising from the evacuation of the water, I had considered this cure as partly the consequence of a low degree of peritoneal inflammation, induced by the stimulus of air admitted through the aperture, or by an extension of the inflammation from the wound, under the circumstances above described. I had therefore proposed to myself the feasibility of imitating this sort of cure, in the first case that occurred favourable for such an attempt.

This, then, was the state of the question when Betty Craine presented herself for a fourth operation:—Was I warranted in trying so hazardous a measure as that of inducing peritoneal inflammation, to cure ascites or dropsy of the ovarium? The degree of success that attends the practice warrants me in premising that I was; and, although the case ultimately terminated fatally, that such practice was by no means the cause of death. At any rate, I consider myself bound to make the case known. I considered the probability of success increased by the improved state of the general health of my patient. Had I attempted it during the weak state my patient was in when I first saw her, and whilst the parietes of the belly were callous and diseased, my expectations would have been disappointed.

Impressed with these considerations, I determined in the present instance to keep the wound open; and for this purpose the cannula was left in the wound for the first night after the operation, secured by means of adhesive plaster, but in such a manner as to admit a free passage through it.

July 25th.—Passed a very tolerable night, and has no complaint to make this morning. The cannula slipped from the wound about six o'clock. It caused a little local irritation, but little or no general pain or uneasiness. A good deal of water has oozed out during the night. A tent of prepared sponge was introduced into the wound, secured by a ligature fixed by adhesive plaster. Bowels to be kept open.

26th.—Patient has got up, and is walking about the room. Tent still continues in the wound, without occasioning pain or tenderness. A good deal more water has oozed out into the bed. Urine not complained of as deficient; skin soft, pulse moderate; no other remarkable symptom.

Two days after this, the tent slipped out of the wound; and it had closed before I had an opportunity of re-introducing it. Having consigned the case to the care of Mr. Watts, she had gone home without my knowledge; most probably from some hyperbolical representation of the danger of the practice.

After this period, the treatment of the case was confined to nourishing regimen and exercise, in so far as that could be attained in her circumstances; and a frequent use of the pulvis jalapæ compositus.

September 25, 1823.—Has occasionally called for a supply of her medicine. To-day (two months after the last operation) she reports herself quite well. No re-accumulation of the water has taken place; health and strength continue to improve; and she has experienced a return of the menstrual discharge, which has been absent for nearly three years. Walks into Douglas and back again, a distance of seven miles, the same day, with ease.

In Mr. Holford Watts's notes of the case, I find the following remark:—"From the foregoing statement, the tent has fully answered our expectations, in altering the nature of the secretions of the cavity, and producing a radical cure of the disease. The tent acted so insensibly in producing this alteration, that we almost despaired of success; but the event has proved the utility of the method of cure practised." Without expressing quite so much confidence in the *modus operandi* in the cure of this case, I shall proceed to detail the subsequent history of it.

August 30th, 1824.—Reports that, till within the last three months, she continued quite well, and menstruated regularly for a period of about nine months. But the disease has lately been increasing rapidly, for which relapse she can assign no cause, excepting a partial exposure to damp. On this occasion the disease is accompanied with much more fever and irritability than at any former one. Has been confined to her bed for the last month, and is not able to sit up for any length of time, owing to the tenderness of the abdomen, and the great weight she has to support. Has taken some of the medicine she formerly found useful, but without any benefit. Pulse 100, and small; respiration very difficult, though it does not amount to orthopnœa, and is most easy in the recumbent posture. Expects no relief from any means except the operation, which I immediately proceeded with, and in the usual manner evacuated twenty-seven quarts of a very dark-coloured liquid, of great weight, from the quantity of mucus with which it was loaded. It was nearly as thick as the white of an egg; the colour reddish, and partaking of that of the grounds of coffee. After standing a night, it deposited a similar sediment.

Towards the close of the tapping, I found it was impossible to evacuate the whole of the fluid from the abdominal cavity, on account of a large tumor of a solid nature, that appeared to occupy the whole of the lower part of the abdomen, in a line with the superior processes of the ossa ilia, and which seemed to obtrude itself against the mouth of the cannula. Pulse 120 after the operation, which she bore remarkably well.

September 1st.—Passed two good nights. There is a little discharge from the puncture. Complains of nothing in particular; but the respiration is still hurried, and the emaciation and varicose state of the superficial veins of the abdomen more conspicuous than ever.

6th.—Wound not yet healed, but discharges very little. There appears to be a fresh effusion of dropsical fluid going on; for I cannot now feel distinctly the tumor in the lower part of the belly. Is able to sit up a little.

October 19th.—Abdominal tumefaction has regained its usual magnitude. Debility and emaciation greater than ever; pulse 120, and small. She is feverish and exhausted; has appetite only for a little tea and toast, and such like. Considered the case desperate, but, at her own request, tapped her again to-day, and drew off sixteen quarts of the same mucous fluid, but mixed with a flocculent matter of a purulent sort.

23d.—Febrile symptoms and dyspnœa considerably abated. The puncture, which was made through the umbilical ring, through which the water protruded, has closed. Bowels to be kept open with the pilulæ rhei co., and no other medicine to be taken.

December 20th.—The dropsical swelling is apparently as large as before it was last evacuated; but she herself thinks it is not so oppressively tense. Back ulcerated, from lying on it so long. Pulse 140, and so feeble as to be hardly perceptible. Respiration is surprisingly free, considering the extent of the disease, and the necessity of being always in the recumbent posture. Is anxious to have the operation performed again; but, taking all circumstances into consideration, it is manifest there is great risk of her sinking suddenly under it. The pilulæ rhei comp., to keep the bowels open, and adhesive plaster to dress the back, were therefore ordered; and a little patience recommended in a cheerful manner.

December 25th.—Died yesterday at five A.M. Having obtained permission, I proceeded to open the body to-day at ten o'clock, accompanied by Mr. Watts, and Mr. Anderson, surgeon, (at that time on a visit here.) Some gangrenous spots appeared on the most prominent parts of the abdomen; and the inferior extremities, especially the right leg, were œdematous. The fluid was, as a first step, evacuated by tapping, but the discharge of it was much impeded by the tumor in the pelvis formerly noticed: we had, therefore, to complete it by laying open the abdomen entirely, when from three to four gallons in all were collected. The whole of the internal cavity of this vast sac was lined with a dense, white, and rough-looking membrane, of a fragile and diseased structure. The abdominal muscles had almost entirely disappeared, so that externally the membrane adhered loosely to a thin layer of blackish cellular membrane, interposed between it and the skin. It adhered also to the diaphragm, and to the liver; both of which had been forced deep under the ribs. Behind it, the intestines were found of an unusually small size, and dark leaden colour. The interior surface of the sac was rendered rough by a sort of tubercular elevation, and flakes of purulent albumen. In general, it was one-eighth of an inch in thickness, and in some places it seemed to consist of more layers than one. On tracing it into the pelvis, its internal surface became loaded with a tubercular, carcinomatous, and pale-coloured fungus, possessing a structure not unlike that of the placenta, and from half an inch to one inch and a half in thickness, especially on the right side, where the punctures had been most frequently made. This was the hard tumor felt occupying the pelvis in a line with the superior processes of the ossa ilii. Tracing the sac still lower, it was discovered to be the cyst of a dropsy, originating in the right ovarium, at the fundus of the sac, or, more properly speaking, its neck. It terminated in a space about the size of the palm of the hand, which had once been occupied by the structures connected with the ovarium; and at this site it involved congeries of several groupes of cysts, like hydatids, of sizes from that of a small orange to that of a filbert-nut, which were smooth and pellucid, and contained a limpid serum. The left ovary was also diseased, and consisted of a congeries of smaller-sized cysts, cemented by a membraneous and fleshy envelope, and exhibiting a beautiful specimen of incipient dropsy of the ovarium, as described by Dr. Baillie, in his Morbid Anatomy; where he views such a congeries, not as hydatids, but as cysts or capsules, which undergo enlargement successively.

The uterus was healthy, and of the usual size. There was a stricture in the rectum, a little below the symoid flexure of the colon; and above it that intestine was much loaded. The liver was half its natural size; but, excepting the diseased look caused on its surface by the adhesion of the sac, it was free from derangement of structure. The gall-bladder was quite distended with black and viscid bile, and filled with biliary concretions. Excepting a most contracted and diminutive size, the contents of the thorax presented nothing remarkable.

The foregoing statement involves four facts and questions of considerable importance in pathology. 1st. The great quantity of fluids evacuated in so short a space of time: no less than ninety-six quarts in eight months, by four operations; and fifty-nine quarts from August to December, 1824, (or four months,) by three. 2dly. The variety in the nature, consistence, and colour of these fluids. 3dly. The possibility of curing ascites and dropsy of the ovaria, by exciting inflammation in the abdominal sac, either by the admission of air into it, or by mechanical irritation. And 4thly. The possibility of a thickening of the parietes of the abdomen by inflammation, or by an exudation of a carcinomatous sort, being mistaken for a tumor rising out of the pelvis. But, as most men will form their own opinions on these facts, I think it preferable to confine myself to simply pointing them out.

*Douglas, Isle of Man;*

*February,* 1826.

